# A crowdsourced intervention to promote hepatitis B and C testing among men who have sex with men in China: study protocol for a nationwide online randomized controlled trial

**DOI:** 10.1186/s12879-018-3403-3

**Published:** 2018-09-29

**Authors:** Thomas Fitzpatrick, Kali Zhou, Yu Cheng, Po-Lin Chan, Fuqiang Cui, Weiming Tang, Katie R Mollan, Wilson Guo, Joseph D Tucker

**Affiliations:** 1 School of Medicine, University of Washington, Seattle, WA USA; 2 Department of Gastroenterology and Hepatology, University of California San Francisco, San Francisco, USA; 3 School of Sociology and Anthropology, Sun Yat-sen University, Guangzhou, China; 4 Division of Communicable Disease, World Health Organization Western Pacific Regional Office, Manila, Philippines; 5 Department of Laboratorial Science and Technology, Peking University, Beijing, China; 6 UNC – Project China, University of North Carolina at Chapel Hill, Chapel Hill, NC USA; 7 School of Medicine, University of North Carolina at Chapel Hill, Chapel Hill, NC USA; 8 Gillings School of Global Public Health – Health Policy and Management, University of North Carolina at Chapel Hill, Chapel Hill, NC USA

**Keywords:** Hepatitis B virus (HBV), Hepatitis C virus (HCV), Testing, Men who have sex with men (MSM), Crowdsourcing, China

## Abstract

**Background:**

The World Health Organization recommends all men who have sex with men (MSM) receive Hepatitis B Virus (HBV) and Hepatitis C Virus (HCV) testing. MSM in China are a high-risk group for HBV and HCV infection, but test uptake is low. Crowdsourcing invites a large group to solve a problem and then shares the solution with the public. This nationwide online randomized controlled trial will evaluate the effectiveness of a crowdsourced intervention to increase HBV and HCV testing among MSM in China.

**Methods:**

Seven hundred MSM will be recruited through social media operated by MSM organizations in China. Eligible participants will be born biologically male, age 16 years or older, report previous anal sex with another man, and reside in China. After completing a baseline online survey, participants will be randomly assigned to intervention or control arms with a 1:1 allocation ratio. The intervention will include two components: (1) a multimedia component will deliver two videos and two images promoting HBV and HCV testing developed through a crowdsourcing contest in China; (2) a participatory component will invite men to submit suggestions for how to improve crowdsourced videos and images. The control arm will not view any images or videos and will not be invited to submit suggestions. All participants will be offered reimbursement for HBV and HCV testing costs. The primary outcome is HBV and HCV test uptake confirmed through electronic submission of test report photos within four weeks of enrolment. Secondary outcomes include self-reported HBV and HCV test uptake, HBV vaccination uptake, and change in stigma toward people living with HBV after four weeks. Primary and secondary outcomes will be calculated using intention to treat and as-exposed analyses and compared using two-sided 95% confidence intervals.

**Discussion:**

Few previous studies have evaluated interventions to increase HBV and HCV testing in middle-income countries with a high burden of hepatitis. Delivering a crowdsourced intervention using social media is a novel approach to increasing hepatitis testing rates. HBV and HCV test uptake will be confirmed through test report photos, avoiding the limitations of self-reported testing outcomes.

**Trial registration:**

NCT03482388 (29 March 2018).

**Electronic supplementary material:**

The online version of this article (10.1186/s12879-018-3403-3) contains supplementary material, which is available to authorized users.

## Background

Hepatitis B Virus (HBV) and Hepatitis C Virus (HCV) are substantial contributors to global morbidity and mortality, together accounting for more deaths annually than HIV [1]. Most individuals affected by chronic viral hepatitis are in low- and middle-income countries (LMIC) [2]. China is a middle-income country with a high burden of hepatitis [3]. HBV and HCV prevalence among the general population in China is 7% and 0.8%, respectively, and China alone has more persons living with chronic viral hepatitis than Europe and the Americas combined [2–4].

Men who have sex with men (MSM) are a group at increased risk for HBV and HCV infection [5, 6]. Prevalence of HBV and HCV is higher among MSM than the general population in multiple contexts, [7–10] including China [11, 12]. As a result, the World Health Organization (WHO) guidelines recommend all MSM receive HBV and HCV testing at least once [13]. Testing is the key first step in the viral hepatitis care continuum and is necessary for infected persons to be linked to care, initiated on treatment, and to achieve viral suppression or cure. Expedited linkage to the viral hepatitis care continuum can reduce liver-related deaths, [14, 15] and hepatitis testing can identify those susceptible to HBV infection and facilitate vaccination [16].

Despite the importance of testing, HBV and HCV testing rates among MSM in China are low. A 2017 nationwide online survey of MSM in China found 59% had never HCV tested, and among those with no or uncertain HBV vaccination, 62% had never HBV tested [17]. Chinese MSM who had not HBV or HCV tested were also less likely to have received screening for HIV and other sexually transmitted infections (STI) [17]. Novel methods of increasing hepatitis testing and linkage to HIV/STI testing services among MSM are needed.

HBV and HCV testing services can be potentially strengthened using crowdsourcing. Crowdsourcing invites a large group to solve a problem and then shares the solution with the public [18]. This bottom-up approach can spur innovation through collecting and refining a wide-range of creative solutions from a diverse group of contributors [13]. Additionally, crowdsourcing engages communities in the development of responses to important issues, thereby increasing community participation and producing more locally relevant solutions [19, 20].

Crowdsourcing has been used to identify health innovation by many funders, including the United States National Institutes of Health, the English National Health Service, the Bill and Melinda Gates Foundation, and the United States government [21, 22]. Non-inferiority randomized controlled trials (RCTs) have shown that crowdsourcing is a cost-effective tool for increasing HIV testing uptake and is as efficacious as evidence-based health marketing materials [23, 24]. Additionally, social media interventions that involve active participation and content generation, such as crowdsourcing, have been associated with higher rates of HIV and STI testing compared to passive testing campaigns [25].

Few previous studies have evaluated interventions to increase hepatitis testing in LMIC, and none of these have targeted MSM or used a crowdsourcing approach [26]. The purpose of this RCT is to address this gap in the current literature and evaluate the impact of a crowdsourced intervention on HBV and HCV test uptake among MSM in China.

### Objectives

Specific aim 1: To compare confirmed HBV and HCV test uptake as a composite endpoint between MSM randomly assigned to a crowdsourced intervention and MSM randomly assigned to control.

Hypothesis 1: MSM assigned to the crowdsourced intervention will have a higher rate of HBV and HCV test uptake compared to MSM in the control arm.

Specific aim 2: To compare change in stigma toward people living with HBV between MSM randomly assigned to a crowdsourced intervention and MSM randomly assigned to control.

Hypothesis 2: MSM assigned to the crowdsourced intervention will have greater reduction in stigma toward people living with HBV compared to MSM in the control arm.

Specific aim 3: To compare self-reported linkage to clinical visits about hepatitis, HBV vaccination, and HIV/STI testing services between MSM randomly assigned to a crowdsourced intervention and MSM randomly assigned to control.

Hypothesis 3: MSM assigned to the crowdsourced intervention will have improved linkage to clinical visits about hepatitis, HBV vaccination, and HIV/STI testing services compared to MSM in the control arm.

## Methods

### Trial design and timeline

**Fig. 1:**
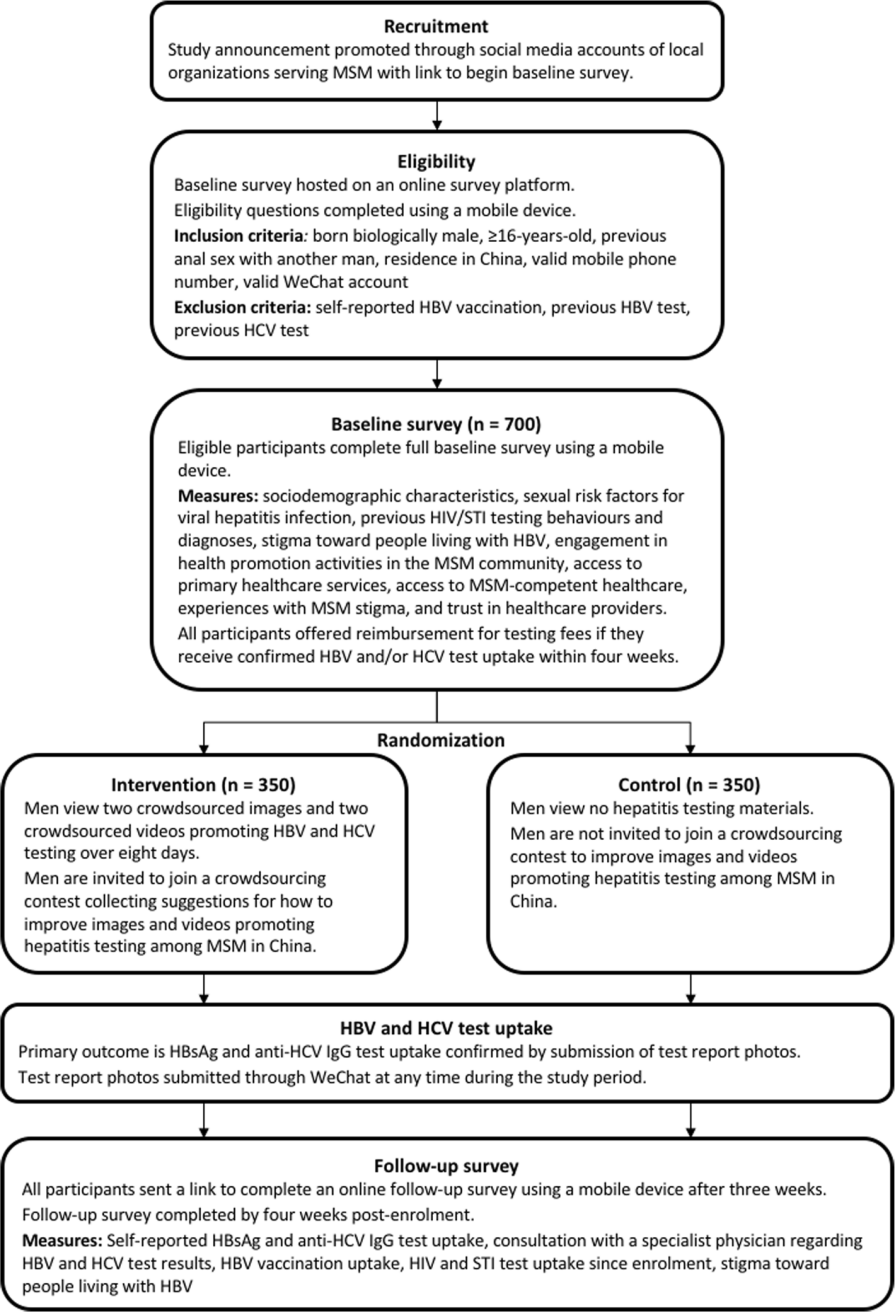
Trial design to evaluate a crowdsourced intervention to increase hepatitis testing among MSM in China

This study will be an online superiority RCT. After completing a baseline survey to assess eligibility, enrolled participants will be randomly assigned in a 1:1 ratio into the intervention or control arms. Men randomly assigned to the intervention arm will be asked to view four crowdsourced images and videos promoting hepatitis testing over 8 days. After viewing each image or video men will be invited to submit suggestions for how to better tailor the crowdsourced materials to the MSM community. Men randomly assigned to the control arm will view no materials and will not be invited to submit suggestions. Participants can submit photos of hepatitis test reports to confirm HBV and HCV test uptake at any time after enrolment. After 3 weeks men will be sent a message asking them to complete a follow-up survey within 1 week. All participants will be assessed for primary and secondary outcomes at 4 weeks (28 days) post-enrolment (Table 1). The study is anticipated to begin enrolment in April 2018 and finish data collection by June 2018. A flowchart summarizing trial design and timeline is presented in Fig. 1.

**Table 1:** Primary and secondary outcomes with definitions

Primary outcome	Definition
Confirmed HBV and HCV test uptake	Defined as men who had both HBsAg test uptake and anti-HCV IgG test uptake confirmed through electronic submission of a test report photo showing serology results, age of tester, sex of tester, and date of test within four weeks of enrolment
Secondary outcomes	
Confirmed HBV test uptake	Defined as men who had HBsAg test uptake confirmed through electronic submission of a test report photo showing serology results, age of tester, sex of tester, and date of test within four weeks of enrolment
Confirmed HCV test uptake	Defined as men who had anti-HCV IgG test uptake confirmed through electronic submission of a test report photo showing serology results, age of tester, sex of tester, and date of test within four weeks of enrolment
Self-reported HBV test uptake	Defined as men who had HBsAg uptake within four weeks of enrolment, self-reported in follow-up survey
Self-reported HCV test uptake	Defined as men who had anti-HCV IgG uptake within four weeks of enrolment, self-reported in follow-up survey
HBV vaccination uptake	Defined as men who had receipt of at least a one dose of the HBV vaccine within four weeks of enrolment, self-reported in follow-up survey
HBV vaccination uptake among men with confirmed susceptibility to HBV infection	Defined as men with negative HBsAg and negative anti-HBs results confirmed through electronic submission of a test report photo showing serology results, who had receipt of at least one dose of the HBV vaccine within four weeks of enrolment, self-reported in follow-up survey
HIV test uptake	Defined as men who had HIV test uptake within four weeks of enrolment, self-reported in follow-up survey
Chlamydia test uptake	Defined as men who had chlamydia test uptake within four weeks of enrolment, self-reported in follow-up survey
Gonorrhoea test uptake	Defined as men who had gonorrhoea test uptake within four weeks of enrolment, self-reported in follow-up survey
Syphilis test uptake	Defined as men who had syphilis test uptake within four weeks of enrolment, self-reported in follow-up survey
Change in stigma toward people living with HBV	Continuous variable, defined as difference between Toronto Chinese HBV Stigma Scale score assessed at follow-up and baseline. Stigma toward people living with HBV will be measured at baseline and follow-up using 20 survey items that are each on a five point Likert scale. The 20 items were originally developed as the Toronto Chinese HBV Stigma Scale (potential range of 20–100), which has been previously validated and correlated to HBV testing behaviours among Chinese populations [39]. Decreased stigma toward people living with HBV will be defined as a mean composite score that is less at follow-up compared to baseline.
Visit with a physician after hepatitis test uptake	Defined as men who had HBV and/or HCV test uptake and saw a physician to discuss hepatitis test results within four weeks of enrolment, self-reported in follow-up survey

### Study setting and recruitment

This study will be conducted online. All recruitment, enrolment, intervention delivery, and data collection will occur through mobile phones and social media smartphone apps. Nearly 70% of the adult population in China owns a smartphone, and 95% of all netizens in China access the internet using a mobile phone [27, 28]. In this study, participants will be recruited through an announcement promoted via social media accounts operated by several local MSM organizations. The announcement will include a link connecting men to the online baseline survey to be completed using a smartphone. The link will remain active until 700 eligible men have enrolled.

Once enrolled, WeChat (Tencent Holdings Limited, Shenzhen, China) will be used to deliver messages and collect confirmatory photos of hepatitis test reports from participants. Additionally, men randomly assigned to the intervention arm will view crowdsourced images and videos through WeChat. WeChat is a multipurpose social media smartphone app with messaging and multimedia functionality. The app had approximately 900 million daily active users in 2017, most of whom live in China, and is commonly used to send and share private messages, social media posts, images, and videos [29].

### Eligibility criteria

Potential participants must first agree to an online informed consent before beginning the baseline online survey. Inclusion criteria are being born biologically male, age 16 years or older, self-reported previous anal sex with another man, and current residence in China. Men who report past HBV vaccination, HBV testing, or HCV testing will be excluded. Those who report uncertain past HBV vaccination, HBV testing, or HCV testing will be considered eligible. All eligible participants must provide a working unique mobile phone number and WeChat account to be enrolled.

### Randomization and allocation

Once enrolled, participants will be assigned in a 1:1 ratio to intervention or control through a randomization procedure using permuted blocks. SAS software (Cary, North Carolina, USA) will be used to create the allocation sequence using the PROC PLAN and RANUNI functions. The allocation sequence will be applied to participants sequentially in the order in which they were enrolled. This RCT is not blinded. Participants will be able to determine their assignment based on whether they received intervention materials during the study period. The primary investigator (TF) will be aware of randomization assignment because intervention materials will be delivered through WeChat.

### Intervention and control

Men randomly assigned to the intervention arm will receive a two-part crowdsourced intervention. The multimedia component of the intervention will deliver two images and two one-minute videos to men through WeChat. One image or video will be delivered every other day after enrolment, for a total of 8 days. Intervention images and videos were developed through a public nationwide crowdsourcing contest conducted in China in 2017.

A steering committee consisting of representatives from 13 national and international organizations was formed to organize a contest to solicit multimedia materials that promote HBV and HCV testing and reduce stigma toward people living with viral hepatitis. An initial public call for images and one-minute videos was promoted through social media and partner organizations. In total, 168 submissions were collected between February and May 2017. All submissions were first evaluated for eligibility and were then reviewed by a judging panel composed of steering committee members. Judges evaluated submissions on a 10-point scale based on each submission’s innovation and creativity, capacity to promote hepatitis testing, and potential to increase social media responsiveness. The twelve highest scoring entries were selected as semi-finalists and provided with feedback for improvement. Semi-finalists then had the opportunity to resubmit their images or videos. Eight submissions (four images and four videos) were selected as contest finalists, with five receiving third place, two receiving second place, and one receiving first place.

To select the finalist entries most appropriate for MSM in China, an online poll was conducted in August 2017. The poll was promoted through local MSM organizations and their social media accounts. Thirty MSM were asked to rank the four finalist videos, and a separate 30 MSM were asked to rank the four finalist images. The two top-ranked images and videos from this poll were selected as materials to be used in the intervention arm of the RCT (intervention images are available in Supplementary Materials as Additional file 1 and Additional file 2).

Because the strength of crowdsourcing includes not only the inflow of creative solutions and new ideas, but also facilitation of active participation and community engagement, the intervention will also include a participatory component. After viewing each image and video, men will be invited to participate in a crowdsourcing contest. Men can submit 50- to 200-word suggestions for how to improve the intervention images and videos to convince more MSM in China to receive hepatitis testing. A contest steering committee composed of hepatitis experts and community representatives will judge each submission on a 10-point scale, and the highest-scoring eight submissions will receive prizes.

Men randomly assigned to control will not view any images or videos and will not be invited to submit ideas for improving hepatitis testing promotional materials. All participants will be informed at enrolment that costs of HBV and HCV testing during the four-week study period can be reimbursed. Because this study is designed as a pragmatic trial with results that will be generalizable to multiple settings, men will be allowed to receive hepatitis testing at any hospital, clinic, or healthcare centre of their choosing.

### Outcomes

HBsAg and anti-HCV IgG test uptake confirmed by test report photo at 4 weeks post-enrolment is the primary outcome of this study. This dichotomous outcome has two component endpoints (HBsAg test uptake and anti-HCV IgG test uptake) that constitute a single composite endpoint. Selection of HBsAg and anti-HCV IgG as serological assays is based on WHO testing guidelines [13]. A participant will only be counted as having the primary outcome if he has submitted a photo to researchers through WeChat or the online follow-up survey that clearly displays tester age, tester sex, test date, and HBsAg and anti-HCV IgG results. If submitted HBV test report photos also include anti-HBs and anti-HBc serological assays, these results will be recorded. Test date must fall within the four-week period (28 days) after enrolment for test uptake to be confirmed.

Confirmed HBsAg and anti-HCV IgG test uptake as independent component endpoints are secondary outcomes. Secondary outcomes also include the following self-reported items on the follow-up survey: HBsAg test uptake, anti-HCV IgG test uptake, HIV test uptake, chlamydia test uptake, gonorrhoea test uptake, syphilis test uptake, visit with specialist physician after HBV or HCV testing, and change in stigma toward people living with HBV during the four-week study period. Self-reported HBV vaccination uptake among all participants and those with confirmed susceptibility to HBV infection (negative anti-HBs and negative HBsAg by test report photo) are also secondary outcomes. Definitions for all study outcomes are provided in Table 1.

### Sample size

A pilot study was conducted in November 2017 to inform sample size calculations. Pilot results are detailed in Supplementary Materials (see Additional file 3). Assuming a confirmed HBV and HCV testing response of 8.5% among intervention participants, 3.0% among control participants, a two-sided significance level of 5%, and 10% not evaluable (e.g., missing data), a total sample size of 674 men would be needed to have 80% power for an exact test. Power was calculated in SAS version 14.2 using the *twosamplefreq* statement in PROC POWER. The total sample size was rounded up to 700 for convenience, which corresponds to approximately 82% power under the above assumptions.

### Data collection and measures

Baseline and follow-up surveys will be delivered to mobile devices and completed online using Wenjuanxing (Sojump, Shanghai, China), a web-based survey tool that meets industry standards for security and functionality. Both surveys were written in Chinese, and 12 local MSM were asked to provide feedback to ensure questions are clear and culturally appropriate.

The baseline survey will first ask respondents to answer questions regarding eligibility criteria. If eligible, respondents will be asked to complete the remainder of the survey. The baseline survey will include the following domains: sociodemographic characteristics, sexual risk factors for viral hepatitis infection, previous HIV/STI testing behaviours and diagnoses, stigma toward people living with HBV, engagement in health promotion activities in the MSM community, access to primary healthcare services, access to MSM-competent healthcare, experiences with MSM stigma in healthcare settings, and trust in healthcare providers. Twenty-eight questions will measure sexual risk factors for viral hepatitis infection, which were adapted from the validated HCV-MOSAIC risk score for HCV infection among MSM [30]. Stigma toward people living with HBV will be assessed using the Toronto Chinese HBV Stigma Scale, a 20-item index validated for Chinese-speaking populations [12]. Engagement in health promotion activities will be measured through six items used in previous evaluations of crowdsourced interventions [31]. Access to MSM-competent healthcare will be assessed through three questions adapted from a recent survey investigating MSM-competent physicians in China [32]. Seven questions adapted from research in the United States will be used to measure experiences with MSM stigma in healthcare settings and trust in healthcare providers [33, 34].

Participants will receive a WeChat message 3 weeks after enrolment asking them to complete a follow-up survey within 1 week. The follow-up survey will collect information regarding the following self-reported outcomes: HBsAg and anti-HCV IgG test uptake, consultation with a specialist physician regarding HBV and HCV test results, HBV vaccination uptake, and HIV and STI test uptake since enrolment. Stigma toward people living with HBV will be reassessed. Participants will be asked to report their exposure to intervention materials and behaviours sharing intervention materials on social media during the four-week study period. Men in the intervention and control arms will receive the same follow-up survey. Men who have not completed the follow-up survey 2 days prior to the end of the four-week study period will be sent a reminder message. Both the baseline and follow-up surveys are expected to take less than 10 min to complete and are available in Supplementary Materials (see Additional file 4 and Additional file 5).

At any time after enrolment and throughout the four-week study period participants can submit photos of their HBV and HCV test reports through WeChat. Test report photos can also be uploaded along with the follow-up survey. The following information will be extracted and recorded from submitted photos: tester age, tester sex, date of test, and test result. No other personally identifiable information will be extracted. Mobile phone numbers and WeChat accounts will be used to link information extracted from test report photos to baseline and follow-up survey responses.

Among men assigned to the intervention arm, suggestions for improving crowdsourced images and videos will be collected through a web-based survey form hosted on Wenjuanxing. Information on contest participation will be linked to baseline and follow-up survey responses using mobile phone numbers and WeChat accounts.

### Confidentiality

Survey data will be collected through and stored on a secure online survey platform (Wenjuanxing). Photos of test reports will be collected through a secure online messaging platform (WeChat) or a secure online survey platform (Wenjuanxing). Participants will be instructed to cover or obscure their name and other personal identifying information on the test report to protect their privacy. Data will be transmitted using 128-bit encryption across the internet and mobile data networks. Responses to both baseline and follow-up surveys, including participant mobile phone numbers and WeChat account numbers, will be stored on a secure server which can be accessed with login information known only to the research team.

### Incentives and reimbursement

Participants who complete the initial survey will receive 4.73 USD (30 RMB), and those who complete the follow-up survey will receive an additional 7.89 USD (50 RMB). All testing costs up to 11.04 USD (70 RMB) for HBV and 6.31 USD (40 RMB) for HCV will be reimbursed to men who submit photos of their test results. Reimbursement amounts are based on prices of national standard HBV testing items (qualitative HBsAg, Anti-HBs, and Anti-HBc serological assay) and HCV testing items (qualitative anti-HCV IgG serological assay), respectively.

Men in the intervention arm who submit suggestions for improving hepatitis testing promotion materials will be eligible to receive contest prizes. Prizes of 78.88 USD (500 RMB), 31.55 USD (200 RMB), and 15.77 USD (100 RMB) will be distributed to one first-place finalist, two second-place finalists, and five third-place finalists, respectively. All incentives and reimbursements will be distributed as electronic money transfers through WeChat.

### Monitoring

A data monitoring committee will not be formed for this RCT because potential for harm to participants is minimal. If any person feels they have experienced an adverse event or unwanted effect from participating in this RCT, they can withdraw at any time. A telephone number and WeChat account will be provided to participants to contact the primary investigator with questions or concerns.

### Data analysis

#### Primary analysis

Participant baseline characteristics and outcomes will be summarized using descriptive statistics. The primary analysis will evaluate the hypothesis that the crowdsourced intervention is superior to control (i.e. no exposure) in increasing HBV and HCV testing among MSM in China. The proportion of men with confirmed HBsAg and anti-HCV IgG test uptake will be calculated for the intervention and control arms separately using a two-sided binomial 95% CI. The difference in proportions with test uptake will be calculated (intervention – control) with a corresponding Wald 95% CI. A Z-test will also be performed to compare the difference in proportions with test uptake between the intervention and control arms. If the 95% CI for the difference in probabilities is entirely above zero, then the intervention will be declared superior to control. If testing proportions are lower than anticipated such that statistical assumptions of the Wald CI are not met, then an exact 95% CI for the difference in probabilities will be used.

The effect of the intervention on HBV and HCV testing will first be evaluated using an intention to treat analysis that includes men lost to follow-up. Those who are lost to follow-up or do not respond will be counted as having not tested for HBV and HCV. An as-exposed analysis will also be performed that reassigns participants based on their self-reported exposure to the intervention materials and adjusts for potential confounders measured in the baseline survey.

#### Effect measure modification analysis

Effect measure modification analyses will evaluate whether the effect of the intervention on confirmed HBV and HCV testing varied in relation to the following factors measured at baseline: (1) engagement in health promotion activities in the MSM community, (2) access to primary healthcare services, (3) access to MSM-competent healthcare, (4) experiences with MSM stigma in healthcare settings, (5) trust in healthcare providers, and (6) age cohort (16–24 years old, 25–29 years old, and ≥ 30 years old). Age cohorts were selected based on previous epidemiological studies investigating HBV infection and vaccine-associated immunity among MSM in China [12].

#### Missing data plan

Any participants lost to follow-up will be included and assumed to have not achieved the primary and secondary outcomes during the study period. If an outcome is missing for < 15% of participants, analyses will use a complete-case approach. If an outcome is missing for ≥15% of participants, analyses will use multiple imputation.

#### Secondary analyses

The intervention and control arms will also be compared for differences in proportions of men achieving secondary outcomes, including confirmed HBsAg test uptake (independent of anti-HCV IgG test uptake), confirmed anti-HCV IgG test uptake (independent of HBsAg test uptake), self-reported HBsAg and anti-HCV IgG test uptake, HBV vaccination, and HIV and STI test uptake during the four-week study period. The analysis approach will be as described for the primary outcome. Change in stigma toward people living with HBV from baseline to week four will be calculated and compared between the intervention and control arms using a Wilcoxon ranksum test.

## Discussion

This study is unique in terms of setting, population, intervention type, and outcome ascertainment, and will therefore make important contributions to the existing literature. A recent systematic review found that few controlled trials have previously evaluated interventions to increase HBV and HCV test uptake [26]. Only one included study had been conducted in a middle-income country, and none had investigated hepatitis testing among MSM [26]. This RCT will extend our understanding of hepatitis testing interventions by investigating MSM in China, a middle-income country with a high burden of hepatitis.

While crowdsourcing has been used to increase HIV testing and condom use, [23, 24] this innovative type of intervention has not been previously applied to HBV and HCV test promotion. By disseminating test promotion materials through social media and encouraging participants to generate content, this crowdsourced intervention has the potential to reach previously underserved MSM and improve hepatitis testing among a population with poor test uptake. Additionally, social media platforms are frequently used by MSM inside and outside China to share content, including health promotion materials [35]. The results of this trial will therefore have implications for future hepatitis testing campaigns targeting MSM in settings with wide-spread social media utilization.

Finally, past studies of hepatitis testing behaviours among MSM have relied on self-report to ascertain whether men had previously received testing [36–38]. However, the reliability of self-reported HBV and HCV test uptake is unknown and potentially subject to recall, ascertainment, and social acceptability biases. This RCT will confirm HBV and HCV test uptake through electronic submission of test report photos. This novel method of outcome ascertainment will provide researchers with reliable biomarker data by verifying testing uptake through hospital or clinic documentation. Participants will be able to receive testing at any facility of their choosing. Because test uptake will not be limited to a specific region or clinical context, the generalizability of results will be enhanced, and this study will be able to inform the design of future hepatitis testing services across a wide-variety of settings.

## Additional files

Additional file 1: Crowdsourced intervention image one. (JPG 1562 kb)

Additional file 2: Crowdsourced intervention image two. (JPG 2308 kb)

Additional file 3: Pilot study results summary. (DOCX 109 kb)

Additional file 4 Baseline survey. (DOCX 49 kb)

Additional file 5 Follow-up survey. (DOCX 42 kb)

## Abbreviations

HBV: Hepatitis B Virus
HCV: Hepatitis C Virus
HIV: Human Immunodeficiency Virus
LMIC: Low- and Middle-Income Countries
MSM: Men Who Have Sex with Men
RCT: Randomized Controlled Trial
STI: Sexually Transmitted Infection
WHO: The World Health Organization
