# Supplementary figures and images for: A crowdsourced intervention to promote hepatitis B and C testing among men who have sex with men in China: study protocol for a nationwide online randomized controlled trial
Source: BMC Infect Dis. 2018 Sep 29;18:489. doi: 10.1186/s12879-018-3403-3 (PMC6162889; doi:10.1186/s12879-018-3403-3)

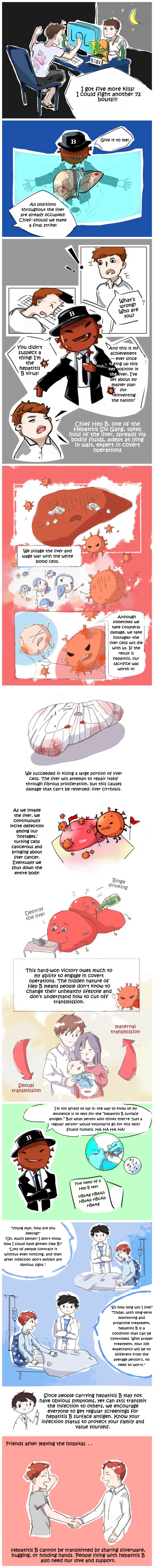

Supplement: Supplementary file 1 — Crowdsourced intervention image one. (JPG 1562 kb) [file 12879_2018_3403_MOESM1_ESM.jpg]

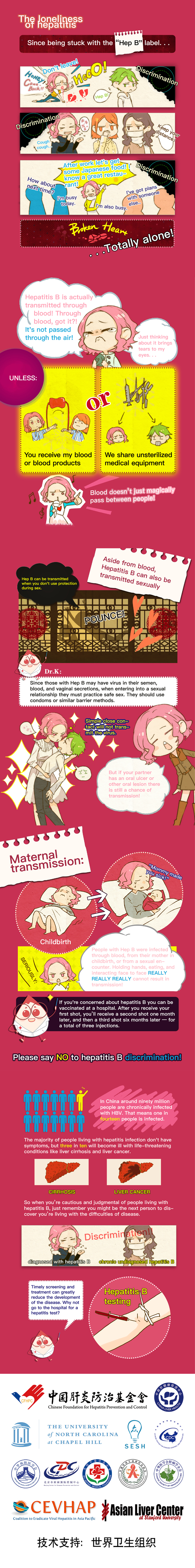

Supplement: Supplementary file 2 — Crowdsourced intervention image two. (JPG 2308 kb) [file 12879_2018_3403_MOESM2_ESM.jpg]
