# Supplementary material for: A crowdsourced intervention to promote hepatitis B and C testing among men who have sex with men in China: study protocol for a nationwide online randomized controlled trial
Source: BMC Infect Dis. 2018 Sep 29;18:489. doi: 10.1186/s12879-018-3403-3 (PMC6162889; doi:10.1186/s12879-018-3403-3)
Supplement: Supplementary file 3 — Pilot study results summary. (DOCX 109 kb) [file 12879_2018_3403_MOESM3_ESM.docx]

**Pilot study results summary**

To inform the development of this study, the multimedia and participatory components of the intervention were evaluated through pilot studies conducted in November 2017 and February 2018, respectively.

In November 2017, 65 eligible MSM were recruited through social media and were offered reimbursement for hepatitis testing costs if they submitted test report photos confirming HBV and HCV test uptake within four weeks. Thirty-four men were assigned to view the four intervention images and videos, and 31 men were assigned to view no materials. 8.8% (3/34) of men in the intervention group had confirmed HBV and HCV testing while 0% (0/31) of men in the control group had confirmed HBV and HCV testing four weeks after enrollment. 9.2% (6/65) of men did not complete the follow-up survey. These results were used as a basis to calculate the sample size necessary to adequately power this full-sized trial. Because we expect the rate of HBV and HCV testing to be higher than 0% in this nationwide study, a conservative estimate of 3.0% HBV and HCV test uptake in the control arm was assumed for sample size calculations.

In February 2018, 59 eligible MSM were recruited through social media and were invited to submit suggestions for how to improve crowdsourced videos and images. To incentivize participation, cash prizes were offered to highest-scoring entries. The first 30 participants were allowed to submit suggestions with no minimum word limit. 80.0% (24/30) of men submitted an entry. However, because most submissions were exceptionally brief (under ten words) and lacked sufficient detail, only one entry was deemed eligible for judging. Researches subsequently adjusted the online submission form to require all suggestions be at least 50 words in length. Another 29 participants were then invited to submit suggestions. After assigning a minimum word limit, 72.4% (21/29) of men submitted an entry, 20 of which were deemed eligible for judging because they described improvements to intervention materials in adequate detail. Consequently, the participatory component of this trial’s crowdsourced intervention will employ a minimum 50-word limit to encourage the submissions of higher quality suggestions.
