# Supplementary material for: A crowdsourced intervention to promote hepatitis B and C testing among men who have sex with men in China: study protocol for a nationwide online randomized controlled trial
Source: BMC Infect Dis. 2018 Sep 29;18:489. doi: 10.1186/s12879-018-3403-3 (PMC6162889; doi:10.1186/s12879-018-3403-3)
Supplement: Supplementary file 4 — Baseline survey. (DOCX 49 kb) [file 12879_2018_3403_MOESM4_ESM.docx]

**Baseline Survey (English)**

1. What was your sex at birth?
   1. Male
   2. Female (end survey)
2. Are you currently 16 years old or older?
   1. Yes
   2. No (end survey)
3. Have you ever had anal sex with another man?
   1. Yes
   2. No (end survey)
4. Have you ever received the HBV vaccination series (the full series requires 3 shots)?
   1. Yes (end survey)
   2. No
   3. Unsure
5. Have you ever been tested for HBV?
   1. Yes (end survey)
   2. No
   3. Unsure
6. Have you ever been tested for HCV?
   1. Yes (end survey)
   2. No
   3. Unsure
7. In which province do you currently reside?
   1. [text]
8. What is your phone number? (We will only use this information for the purposes of this research project. We will not distribute your phone number to any agency or individual.)
   1. [number]
9. What is your WeChat ID? (We will only use this information for the purposes of this research project. We will not distribute your WeChat account to any agency or individual.)
   1. [WeChat ID]

The following questions will ask you about sociodemographic characteristics.

1. How old are you?
   1. [years]
2. Are you currently living in an urban or rural area?
   1. Urban
   2. Rural
3. What kind of hukou household registration do you currently hold?
   1. Urban
   2. Rural
4. What is your highest level of education?
   1. High school or below
   2. Technical school
   3. Some college or a bachelors degree
   4. Masters, PhD, or above
5. What is your main occupation?
   1. Student
   2. Farmer
   3. Civil servant
   4. Labor worker (blue collar)
   5. Office worker (white collar)
   6. Sex worker
   7. Service/retail
   8. Unemployed
   9. Other
6. What is your monthly individual income (after taxes) from all sources?
   1. Less than 1500 RMB
   2. 1500 – 3000 RMB
   3. 3001 – 5000 RMB
   4. 5001 – 8000 RMB
   5. Greater than 8000
7. What is your marital status (referring to woman)?
   1. Never married
   2. Engaged or married
   3. Separated or divorced
   4. Widowed
8. What is your gender identity?
   1. Male
   2. Female
   3. Transgender
   4. Unsure/Other
9. What is your sexual orientation?
   1. Homosexual
   2. Bisexual
   3. Heterosexual
   4. Unsure/other

The following questions will ask you about your experiences with healthcare providers.

1. How long ago was the last time you saw a doctor?
   1. Within the past 6 months
   2. Within the past year
   3. Within the past 2 years
   4. Within the past 5 years
   5. More than 5 years ago
   6. I have never seen a doctor
2. How long ago was the last time you saw a doctor for issues related to sexual health?
   1. Within the past 6 months
   2. Within the past year
   3. Within the past 2 years
   4. Within the past 5 years
   5. More than 5 years ago
   6. I have never seen a doctor for issues related to sexual health
3. Regarding your latest experience seeing a doctor, where were you seen?
   1. Hospital
   2. Community health center
   3. Private clinic
   4. Other
4. Have you ever seen a doctor or received healthcare services at a community health center?
   1. Yes
   2. No
5. Do you currently have an established regular doctor or primary care provider (by primary care doctor, we mean a trusted, non-specialist that you see on a regular basis)?
   1. Yes
   2. No
6. Have you ever heard of HBV before?
   1. Yes
   2. No
7. Have you ever heard of HCV before?
   1. Yes
   2. No
8. Has a doctor ever recommended that you be tested for HBV?
   1. Yes
   2. No
9. Has a doctor ever recommended that you be tested for HCV?
   1. Yes
   2. No
10. Has a doctor ever asked you about having sex with other men?
    1. Yes
    2. No
11. Has a doctor ever asked you about anal sex?
    1. Yes
    2. No
12. Has a doctor ever suggested you receive HIV or syphilis testing?
    1. Yes
    2. No

The following questions refer to your past experiences with doctors. Please indicate if you agree or disagree with the following statements.

1. I have been mistreated by doctors because of my sexual orientation.
   1. Strongly disagree
   2. Disagree
   3. Neither agree or disagree
   4. Agree
   5. Strongly agree
2. I have been ignored by doctors because of my sexual orientation.
   1. Strongly disagree
   2. Disagree
   3. Neither agree or disagree
   4. Agree
   5. Strongly agree
3. My healthcare isn’t as good as others’ because of my sexual orientation.
   1. Strongly disagree
   2. Disagree
   3. Neither agree or disagree
   4. Agree
   5. Strongly agree
4. I am comfortable telling my doctor I have sex with men.
   1. Strongly disagree
   2. Disagree
   3. Neither agree or disagree
   4. Agree
   5. Strongly agree
5. I trust that healthcare providers are giving me the best treatment available.
   1. Strongly disagree
   2. Disagree
   3. Neither agree or disagree
   4. Agree
   5. Strongly agree
6. I trust that healthcare providers have my best interest in mind when treatment me.
   1. Strongly disagree
   2. Disagree
   3. Neither agree or disagree
   4. Agree
   5. Strongly agree
7. I trust that healthcare providers will tell me if a mistake is made about my medical treatment.
   1. Strongly disagree
   2. Disagree
   3. Neither agree or disagree
   4. Agree
   5. Strongly agree

The following questions will ask about your feelings and attitudes regarding HBV. Please indicate whether you agree or disagree with the following statements.

1. People with Hepatitis B should be isolated from others to protect the public.
   1. Strongly disagree
   2. Disagree
   3. Neither agree nor disagree
   4. Agree
   5. Strongly agree
2. It is not safe for people with Hepatitis B to work with children.
   1. Strongly disagree
   2. Disagree
   3. Neither agree nor disagree
   4. Agree
   5. Strongly agree
3. People with Hepatitis B should not be allowed to work in certain areas such as restaurants.
   1. Strongly disagree
   2. Disagree
   3. Neither agree nor disagree
   4. Agree
   5. Strongly agree
4. I would feel pity for someone with Hepatitis B.
   1. Strongly disagree
   2. Disagree
   3. Neither agree nor disagree
   4. Agree
   5. Strongly agree
5. A person with Hepatitis B must have done something wrong and deserves to be sick.
   1. Strongly disagree
   2. Disagree
   3. Neither agree nor disagree
   4. Agree
   5. Strongly agree
6. Parents are at fault for their children getting Hepatitis B.
   1. Strongly disagree
   2. Disagree
   3. Neither agree nor disagree
   4. Agree
   5. Strongly agree
7. People with Hepatitis B should be ashamed of their illness.
   1. Strongly disagree
   2. Disagree
   3. Neither agree nor disagree
   4. Agree
   5. Strongly agree
8. People with Hepatitis B are unclean.
   1. Strongly disagree
   2. Disagree
   3. Neither agree nor disagree
   4. Agree
   5. Strongly agree
9. I would not want my child to attend school where one of the students had Hepatitis B.
   1. Strongly disagree
   2. Disagree
   3. Neither agree nor disagree
   4. Agree
   5. Strongly agree
10. I would not want to work in an office where one of the people there had Hepatitis B.
    1. Strongly disagree
    2. Disagree
    3. Neither agree nor disagree
    4. Agree
    5. Strongly agree
11. I would not want to go to a small neighborhood grocery store where the owner had Hepatitis B.
    1. Strongly disagree
    2. Disagree
    3. Neither agree nor disagree
    4. Agree
    5. Strongly agree
12. I would feel uncomfortable wearing a sweater once worn by a person with Hepatitis B.
    1. Strongly disagree
    2. Disagree
    3. Neither agree nor disagree
    4. Agree
    5. Strongly agree
13. I would feel uncomfortable sharing a meal with someone who has Hepatitis B.
    1. Strongly disagree
    2. Disagree
    3. Neither agree nor disagree
    4. Agree
    5. Strongly agree
14. I would not want to be friends with someone with Hepatitis B.
    1. Strongly disagree
    2. Disagree
    3. Neither agree nor disagree
    4. Agree
    5. Strongly agree
15. I would not employ someone with Hepatitis B to work for me.
    1. Strongly disagree
    2. Disagree
    3. Neither agree nor disagree
    4. Agree
    5. Strongly agree
16. I would feel uncomfortable having a conversation with someone who had Hepatitis B.
    1. Strongly disagree
    2. Disagree
    3. Neither agree nor disagree
    4. Agree
    5. Strongly agree
17. I would not kiss someone with Hepatitis B.
    1. Strongly disagree
    2. Disagree
    3. Neither agree nor disagree
    4. Agree
    5. Strongly agree
18. I would not date someone with Hepatitis B.
    1. Strongly disagree
    2. Disagree
    3. Neither agree nor disagree
    4. Agree
    5. Strongly agree
19. I would not marry someone with Hepatitis B.
    1. Strongly disagree
    2. Disagree
    3. Neither agree nor disagree
    4. Agree
    5. Strongly agree
20. I would avoid rooming with someone with Hepatitis B.
    1. Strongly disagree
    2. Disagree
    3. Neither agree nor disagree
    4. Agree
    5. Strongly agree

The following questions are designed to measure the level of your engagement with the MSM community. It is not a test. There are no right or wrong answers.

1. Are you aware of any ongoing community events promoting HIV, hepatitis, or STI testing among MSM?
   1. Yes
   2. No
2. Have you ever volunteered at a health clinic or other location that provided sexual health services among MSM?
   1. Yes
   2. No
3. Have you ever helped organize a testing and/or awareness campaign (e.g. HIV, condom use) that promoted sexual health among MSM?
   1. Yes
   2. No
4. Have you ever encouraged someone else to get tested for HIV, hepatitis, or another sexually transmitted disease?
   1. Yes
   2. No
5. Have you ever accompanied a friend or partner to a testing facility to get tested for HIV, hepatitis, or another sexually transmitted disease?
   1. Yes
   2. No
6. Have you ever participated in online forums or discussions on social media about HIV, hepatitis, or STD testing or related services?
   1. Yes
   2. No

The following questions will ask you about your sexual history.

1. Approximately how many male partners have you had sex with in the past 12 months?
   1. [number]
2. What best describes your typical sexual position when having sex?
   1. Insertive
   2. Mostly insertive
   3. Insertive/receptive
   4. Mostly receptive
   5. Receptive
3. Did you use a condom the last time you had sex?
   1. Yes
   2. No
4. Have you ever had condomless receptive anal intercourse?
   1. Yes
   2. No
5. Have you had condomless receptive anal intercourse in the past 6 months?
   1. Yes
   2. No
6. Have you ever shared sex toys?
   1. Yes
   2. No
7. Have you shared sex toys in the past 6 months?
   1. Yes
   2. No
8. Have you ever received unprotected anal fisting?
   1. Yes
   2. No
9. Have you received unprotected anal fisting in the past 6 months?
   1. Yes
   2. No
10. Have you ever participated in group sex?
    1. Yes
    2. No
11. Have you participated in group sex in the past 6 months?
    1. Yes
    2. No
12. Which of the following drugs have you consumed prior to sex?
    1. None
    2. Poppers (Rush)
    3. Ecstasy
    4. Meth
    5. Ketamine
    6. Cocaine
    7. Other:
13. Which of the following drugs have you consumed prior to sex in the past 12 months?
    1. None
    2. Poppers (Rush)
    3. Ecstasy
    4. Meth
    5. Ketamine
    6. Cocaine
    7. Other:
14. Have you shared a straw or other tools to consume drugs in the past 12 months?
    1. Yes
    2. No
15. Have you ever injected drugs?
    1. Yes
    2. No
16. What drugs have you injected?
    1. Heroin
    2. Meth
    3. Cocaine
    4. Other:
17. Have you injected drugs in the past 12 months?
    1. Yes
    2. No

The following questions will ask you about HIV and STI testing.

1. Have you ever been tested for HIV (facility or self-testing)?
   1. Yes – I’ve tested and am HIV positive
   2. Yes – I’ve tested and am HIV negative
   3. Yes – I never got my test results
   4. No – I have never been tested for HIV
2. Have you ever had a blood test for syphilis?
   1. Yes
   2. No
3. Have you ever been diagnosed with syphilis?
   1. Yes
   2. No
4. Were you diagnosed with syphilis in the past 12 months?
   1. Yes
   2. No
5. Have you ever been tested for chlamydia?
   1. Yes
   2. No
6. Have you ever been diagnosed with chlamydia?
   1. Yes
   2. No
7. Were you diagnosed with chlamydia in the past 12 months?
   1. Yes
   2. No
8. Have you ever been tested for gonorrhea?
   1. Yes
   2. No
9. Have you ever been diagnosed with gonorrhea?
   1. Yes
   2. No
10. Were you diagnosed with gonorrhea in the past 12 months?
    1. Yes
    2. No

**Baseline Survey (Chinese)**

A1. 请问你出生时的生理性别是?？

1. 男性
2. 女性（终止调查）

A2. 请问你的年龄是16岁或以上吗？

1. 是的
2. 没有 （终止调查）

A3. 请问你和其他男性有过肛交行为吗？

1. 有过
2. 没有（终止调查）

A4. 请问你曾经接种过全套乙肝疫苗吗? (全套疫苗包含三针)？

1. 有过（终止调查）
2. 没有
3. 不清楚

A5. 请问你检测过乙肝吗?？

1. 有过（终止调查）
2. 没有
3. 不确定

A6. 请问你检测过丙肝吗?

1. 有过（终止调查）
2. 没有
3. 不清楚

A7. 你目前所在的省份或地区？

a.（省份）

A8. 请问你的手机号码是多少？
（添加手机号码的目的仅为发放奖励与进行后续调查；整个过程中，我们都会严格保障参与者的隐私。）

a.（号码）

A9. 请问你的微信账号（非微信昵称）是多少？

（添加微信的目的仅为发放奖励与进行后续调查。我们的微信账号由一名主要研究者操作，其已接受严格的研究伦理培训；整个过程中，我们都会严格保障参与者的隐私。）

a.（账号）

以下问题将询问你的社会人口背景信息

B1. 请问你的年龄是？

（年龄）

B2. 请问你目前住在城市还是农村地区？

1. 城市
2. 农村

B3.请问你目前的户口是？

1. 城市户口
2. 农村户口

B4. 请问你最高受教育程度是？

1. 高中或以下
2. 技工或专科学校
3. 本科
4. 硕士、博士或以上

B5. 请问你的主要职业是？

1. 学生
2. 农民
3. 公务员
4. 体力工人（蓝领）
5. 办公室员工（白领）
6. 性工作者
7. 服务/销售
8. 待业、失业
9. 其他

B6. 请问你的月收入(税后)是多少？

1. 少于1500人民币
2. 1500-3000人民币
3. 3001-5000人民币
4. 5001-8000人民币
5. 多于8000人民币

B7. 请问你的婚姻状况是？

1. 未婚
2. 订婚/已婚
3. 离婚/分居
4. 丧偶

B8. 请问你的性别认同？

1. 男性
2. 女性
3. 跨性别者
4. 不确定/其他

B9. 请问你的性取向是

1. 同性恋
2. 双性恋
3. 异性恋
4. 不确定/其他

以下问题将询问你的求医经验

C1. 请问你上一次是何时看医生的？

1. 过去6个月内
2. 过去1年内
3. 过去2年内
4. 过去5年内
5. 5年多以前
6. 从未看过

C2. 请问你上一次看医生时涉及性健康（比如说性行为、性病等）事项的是何时？

1. 过去6个月内
2. 过去1年内
3. 过去2年内
4. 过去5年内
5. 5年多以前
6. 从未看过

C3. 请问你上次看医生时，是在哪里？

1. 医院
2. 社区卫生服务中心／站
3. 私人诊所
4. 其他

C4. 请问你在社区卫生服务中心／站看过医生或者接受过其他医疗保健服务吗？

1. 有
2. 没有

C5. 请问你目前有固定的医生或初级保健医生吗？
（初级保健医生指的是一个在社区层面的、值得信赖的、你定期去看的非专科医生）

1. 有
2. 没有

C6. 请问你以前听说过乙肝吗?？

1. 有
2. 没有

C7. 请问你以前听说过丙肝吗?？

1. 有
2. 没有

C8. 请问是否曾经有医生建议你进行乙肝检测？

1. 有
2. 没有

C9. 请问是否曾经有医生建议你进行丙肝检测？

1. 有
2. 没有

C10. 请问医生有没有问过你，你是否和其他男人发生过性关系？

1. 有
2. 没有

C11. 请问医生有没有问过你关于肛交性行为的问题？

1. 有
2. 没有

C12. 请问医生有没有建议你检测HIV或梅毒？

1. 有
2. 没有

以下问题是关于你长期以来的就医体验，请回答你是否同意以下观点

C13. 因为我的性取向，我受到医生不公平地对待。

1. 完全不同意
2. 不同意
3. 既不同意，也不反对
4. 同意
5. 非常同意

C14. 因为我的性取向，我被医生忽视了。

1. 完全不同意
2. 不同意
3. 既不同意，也不反对
4. 同意
5. 非常同意

C15. 因为我的性取向，我所接受到的医疗服务没别人好。

1. 完全不同意
2. 不同意
3. 既不同意，也不反对
4. 同意
5. 非常同意

C16. 告诉医生我曾经和男性有过性行为，我感到自在。

1. 完全不同意
2. 不同意
3. 既不同意，也不反对
4. 同意
5. 非常同意

C17. 我相信医务人员给我提供了最好的治疗。

1. 完全不同意
2. 不同意
3. 既不同意，也不反对
4. 同意
5. 非常同意

C18. 我相信医务人员在尽最大的努力为我着想。

1. 完全不同意
2. 不同意
3. 既不同意，也不反对
4. 同意
5. 非常同意

C19. 我相信如果在治疗过程中有失误，医务人员会如实告诉我。

1. 完全不同意
2. 不同意
3. 既不同意，也不反对
4. 同意
5. 非常同意

以下问题将询问你对于乙肝检测的态度和经历，请回答你是否同意以下观点

D1. 为了保护大众，乙肝感染者应被隔离。

1. 非常不同意
2. 不同意
3. 既不同意也不反对
4. 同意
5. 非常同意

D2. 如果乙肝感染者从事与儿童密切接触的工作，他们可能会给儿童带来感染风险。

1. 非常不同意
2. 不同意
3. 既不同意也不反对
4. 同意
5. 非常同意

D3. 乙肝感染者不应被允许在餐馆等场所工作。

1. 非常不同意
2. 不同意
3. 既不同意也不反对
4. 同意
5. 非常同意

D4. 我觉得乙肝感染者很可怜。

1. 非常不同意
2. 不同意
3. 既不同意也不反对
4. 同意
5. 非常同意

D5. 乙肝感染者一定做过不好的事情，被感染是活该的。

1. 非常不同意
2. 不同意
3. 既不同意也不反对
4. 同意
5. 非常同意

D6. 孩子感染乙肝是因为父母的行为失当。

1. 非常不同意
2. 不同意
3. 既不同意也不反对
4. 同意
5. 非常同意

D7. 乙肝感染者应为他们的病痛感到羞耻。

1. 非常不同意
2. 不同意
3. 既不同意也不反对
4. 同意
5. 非常同意

D8. 乙肝感染者不干净。

1. 非常不同意
2. 不同意
3. 既不同意也不反对
4. 同意
5. 非常同意

D9. 如果我孩子的同学里有乙肝感染者，我不想再让孩子去那里上学。

1. 非常不同意
2. 不同意
3. 既不同意也不反对
4. 同意
5. 非常同意

D10. 我不想和乙肝感染者在同一间办公室工作。

1. 非常不同意
2. 不同意
3. 既不同意也不反对
4. 同意
5. 非常同意

D11. 如果隔壁小卖部的老板是乙肝感染者，我不想再去光顾了。

1. 非常不同意
2. 不同意
3. 既不同意也不反对
4. 同意
5. 非常同意

D12. 我穿乙肝感染者穿过的毛衣会觉得不舒服。

1. 非常不同意
2. 不同意
3. 既不同意也不反对
4. 同意
5. 非常同意

D13. 我和乙肝感染者一起吃饭会觉得不舒服。

1. 非常不同意
2. 不同意
3. 既不同意也不反对
4. 同意
5. 非常同意

D14. 我不想和乙肝感染者成为朋友。

1. 非常不同意
2. 不同意
3. 既不同意也不反对
4. 同意
5. 非常同意

D15. 我不想雇佣乙肝感染者。

1. 非常不同意
2. 不同意
3. 既不同意也不反对
4. 同意
5. 非常同意

D16. 我和乙肝感染者谈话会觉得不舒服。

1. 非常不同意
2. 不同意
3. 既不同意也不反对
4. 同意
5. 非常同意

D17. 我不会亲吻乙肝感染者。

1. 非常不同意
2. 不同意
3. 既不同意也不反对
4. 同意
5. 非常同意

D18. 我不会和乙肝感染者谈恋爱。

1. 非常不同意
2. 不同意
3. 既不同意也不反对
4. 同意
5. 非常同意

D19. 我不会和乙肝感染者结婚。

1. 非常不同意
2. 不同意
3. 既不同意也不反对
4. 同意
5. 非常同意

D20. 我会避免和乙肝感染者住在一起。

1. 非常不同意
2. 不同意
3. 既不同意也不反对
4. 同意
5. 非常同意

以下的问题将涉及你在男同社群的参与经验（如男同圈子，社交网络等）。这不是测试，所以答案没有对错

E1. 请问你是否了解任何正在进行的在男同社群内推广HIV、肝炎或性传播疾病检测的活动？

1. 是
2. 否

E2. 请问你是否在男同社群内的健康诊所或其他提供性健康服务的组织做过志愿服务？

1. 是
2. 否

E3. 请问你是否帮忙组织过男同社群内关于HIV检测或相关知识（如艾滋、安全套使用等）的活动以推广性健康？

1. 是
2. 否

E4. 请问你是否鼓励过其他人去做HIV、肝炎或其他性传播疾病的检测？

1. 是
2. 否

E5. 请问你是否陪朋友或者伴侣一起去检查过HIV、肝炎或者其他性传播疾病？

1. 是
2. 否

E6. 请问你是否在社交媒体或网络社区（如微信、微博）上讨论过艾滋、肝炎或其它性传播疾病检测或其他相关的服务？

1. 是
2. 否

下一部分的问卷会询问你和其他男性之间性行为的一些问题。

F1. 请问在过去12个月，你大约和多少位男性发生过性行为？

a.（数字）

F2. 请问发生性行为时，你通常是什么性关系体位？

1. 插入方（做1）
2. 大多是插入
3. 插入式/接受式
4. 大多数接受式
5. 受入方（做0）

F3. 请问在上一次发生性行为时候你使用安全套了吗？

1. 有过
2. 没有

F4. 请问你曾经有过被无套插入的肛交行为吗？

1. 有过
2. 没有

F5. 请问在过去的6个月，你有过被无套插入的肛交行为吗？

1. 有过
2. 没有

F6. 请问你曾经和他人共用过情趣用品吗？

1. 有过
2. 没有

F7. 请问在过去的6个月，你和他人共用过情趣用品吗？

1. 有过
2. 没有

F8. 请问你是否和他人发生过不带套的肛门拳交性行为？

1. 有过
2. 没有

F9. 请问在过去的6个月，你是否和他人发生过不带套的肛门拳交性行为？

1. 有过
2. 没有

F10. 请问你是否参与过群交行为？

1. 有过
2. 没有

F11. 请问在过去的6个月，你是否参与过群交行为？

1. 有过
2. 没有

F12. 请问在发生性行为之前，你曾经服用过下列哪种药物？

1. 未服用过任何药物
2. Rush
3. 摇头丸
4. 冰毒
5. K粉
6. 可卡因
7. 其他

F13. 请问在过去12个月，发生性行为之前，你曾经服用过下列哪种药物？

1. 未服用过任何药物
2. Rush
3. 摇头丸
4. 冰毒
5. K粉
6. 可卡因
7. 其他

F14. 请问在过去12个月，你是否和他人共用过吸管或其他工具来服用药物？

1. 有过
2. 没有

F15. 请问你是否曾经注射过毒-品？

1. 有过
2. 没有

F16. 请问你曾经注射过下列哪种药物？

1. 海洛因
2. 冰毒
3. 可卡因
4. 其他

F17. 请问在过去12个月，你是否曾经注射过毒-品？

1. 有过
2. 没有

以下问题关于HIV和性传播疾病的检测。

G1. 请问你之前检测过HIV吗？（自查或者设施检查）

1. 有过 – 我检测过HIV且感染了
2. 有过 – 我检测过HIV且没有感染
3. 有过 – 我不知道我的检测结果
4. 没有 – 我从来没有检测过HIV

G2. 请问你曾经抽血检测过梅毒吗？

1. 有过
2. 没有

G3. 请问你曾经被确诊过梅毒阳性吗？

1. 是的
2. 没有

G4. 请问在过去12个月，你是否被确诊过梅毒阳性？

1. 是
2. 否

G5. 请问你曾经检测过衣原体吗？

1. 有过
2. 没有

G6. 请问你曾经被确诊过衣原体阳性吗？

1. 是的
2. 没有

G7. 请问在过去12个月，你是否被确诊过衣原体阳性？

1. 是
2. 否

G8. 请问你曾经检测过淋病吗？

a. 有过

b. 没有

G9. 请问你曾经被确诊过淋病阳性吗？

a. 是的

b. 没有

G10. 请问在过去12个月，你是否被确诊过淋病阳性？

a. 是

b. 否
