# Supplementary material for: A crowdsourced intervention to promote hepatitis B and C testing among men who have sex with men in China: study protocol for a nationwide online randomized controlled trial
Source: BMC Infect Dis. 2018 Sep 29;18:489. doi: 10.1186/s12879-018-3403-3 (PMC6162889; doi:10.1186/s12879-018-3403-3)
Supplement: Supplementary file 5 — Follow-up survey. (DOCX 42 kb) [file 12879_2018_3403_MOESM5_ESM.docx]

**Follow-up Survey (English)**

1. What is your phone number? (We will only use this information for the purposes of this research project. We will not distribute your phone number to any agency or individual.)
   1. [number]
2. What is your WeChat ID? (We will only use this information for the purposes of this research project. We will not distribute your WeChat account to any agency or individual.)
   1. [WeChat ID]

The following questions are about HBV testing.

1. Have you been tested for HBV (HBsAg) in the past 3-4 weeks?
   1. Yes
   2. No
2. Would you be willing to submit a photo of your HBV test results to us? (Test results will be kept on a secure server only accessible to one researcher. **Participants will be reimbursed 70 RMB for submitted test results.** Please ensure age, sex, date of test, and test results are clearly visible. **Please cover or obscure your name, patient identification number, and tax identification number on the report to best protect your privacy.**)
   1. Yes (link to submit photo)
   2. No
   3. Already submitted through WeChat
3. In the past 3-4 weeks have you discussed HBV testing with a doctor?
   1. Yes
   2. No
4. Where did you get tested for HBV?
   1. Large Municipal Hospital
   2. Community health center
   3. Private clinic
   4. NGO
   5. CDC office
5. What was the result of the HBV test?
   1. Currently infected with HBV (i.e. HBsAg+)
   2. Previously infected with HBV, now cleared (i.e. Anti-HBc+, Anti-HBs+, HBsAg-)
   3. Previously vaccinated (i.e. Anti-HBs+, HBsAg-)
   4. Susceptible to HBV infection (i.e. Anti-HBs-, HBsAg-)
   5. Unsure
   6. Did not receive my result
6. Did you see a hepatitis specialist physician after receiving your test result?
   1. Yes
   2. No
7. Did you receive a follow-up HBV DNA test?
   1. Yes
   2. No
8. Did you receive at least one dose of the HBV vaccine?
   1. Yes
   2. No
9. Why did you not upload your HBV test results?
   1. I’m concerned about exposing private information
   2. I have not yet received my test results
   3. Test reimbursement is too small
   4. Other
10. Why did you not get HBV tested?
    1. I’m not sure where to get tested
    2. There’s not been enough time to get tested
    3. Transportation to a testing center is inconvenient
    4. Testing fees are too expensive
    5. I don’t think I’m at risk of infection
    6. I’m worried about experiencing discrimination if diagnosed with HBV
    7. I think there are no treatment options for HBV
    8. I don’t think HBV is a serious concern
    9. My doctor wouldn’t let me test for HBV

The following questions are about HCV testing.

1. Have you been tested for HCV (anti-HCV IgG) in the past 3-4 weeks?
   1. Yes
   2. No
2. Would you be willing to submit a photo of your HCV test results to us? (Test results will be kept on a secure server only accessible to one researcher. **Participants will be reimbursed 40 RMB for submitted test results.** Please ensure age, sex, date of test, and test results are clearly visible. **Please cover or obscure your name, patient identification number, and tax identification number on the report to best protect your privacy.**)
   1. Yes (link to submit photo)
   2. No
   3. Already submitted through WeChat
3. In the past 3-4 weeks have you discussed HCV testing with a doctor?
   1. Yes
   2. No
4. Where did you get tested?
   1. Large Municipal Hospital
   2. Community health center
   3. Private clinic
   4. NGO
   5. CDC office
5. What was the result of HCV antibody test?
   1. Positive
   2. Negative
   3. Did not receive my result
   4. Unsure
6. Did you see a hepatitis specialist physician after receiving your test results?
   1. Yes
   2. No
7. Did you receive a confirmatory HCV viral load test?
   1. Yes
   2. No
8. What was the result of your confirmatory HCV viral load test?
   1. Positive
   2. Negative
   3. Unsure
9. Why did you not upload your HCV test results?
   1. I’m concerned about exposing private information
   2. I have not yet received my test results
   3. Test reimbursement is too small
   4. Other
10. Why did you not get HCV tested?
    1. I’m not sure where to get tested
    2. There’s not been enough time to get tested
    3. Transportation to a testing center is inconvenient
    4. Testing fees are too expensive
    5. I don’t think I’m at risk of HCV infection
    6. I’m worried about experiencing discrimination if diagnosed with HCV
    7. I think there are no treatment options for HCV
    8. I don’t think HCV is a serious concern
    9. My doctor wouldn’t let me HCV test

The following questions will ask about your feelings and attitudes regarding HBV. Please indicate whether you agree or disagree with the following statements.

1. People with Hepatitis B should be isolated from others to protect the public.
   1. Strongly disagree
   2. Disagree
   3. Neither agree nor disagree
   4. Agree
   5. Strongly agree
2. It is not safe for people with Hepatitis B to work with children.
   1. Strongly disagree
   2. Disagree
   3. Neither agree nor disagree
   4. Agree
   5. Strongly agree
3. People with Hepatitis B should not be allowed to work in certain areas such as restaurants.
   1. Strongly disagree
   2. Disagree
   3. Neither agree nor disagree
   4. Agree
   5. Strongly agree
4. I would feel pity for someone with Hepatitis B.
   1. Strongly disagree
   2. Disagree
   3. Neither agree nor disagree
   4. Agree
   5. Strongly agree
5. A person with Hepatitis B must have done something wrong and deserves to be sick.
   1. Strongly disagree
   2. Disagree
   3. Neither agree nor disagree
   4. Agree
   5. Strongly agree
6. Parents are at fault for their children getting Hepatitis B.
   1. Strongly disagree
   2. Disagree
   3. Neither agree nor disagree
   4. Agree
   5. Strongly agree
7. People with Hepatitis B should be ashamed of their illness.
   1. Strongly disagree
   2. Disagree
   3. Neither agree nor disagree
   4. Agree
   5. Strongly agree
8. People with Hepatitis B are unclean.
   1. Strongly disagree
   2. Disagree
   3. Neither agree nor disagree
   4. Agree
   5. Strongly agree
9. I would not want my child to attend school where one of the students had Hepatitis B.
   1. Strongly disagree
   2. Disagree
   3. Neither agree nor disagree
   4. Agree
   5. Strongly agree
10. I would not want to work in an office where one of the people there had Hepatitis B.
    1. Strongly disagree
    2. Disagree
    3. Neither agree nor disagree
    4. Agree
    5. Strongly agree
11. I would not want to go to a small neighborhood grocery store where the owner had Hepatitis B.
    1. Strongly disagree
    2. Disagree
    3. Neither agree nor disagree
    4. Agree
    5. Strongly agree
12. I would feel uncomfortable wearing a sweater once worn by a person with Hepatitis B.
    1. Strongly disagree
    2. Disagree
    3. Neither agree nor disagree
    4. Agree
    5. Strongly agree
13. I would feel uncomfortable sharing a meal with someone who has Hepatitis B.
    1. Strongly disagree
    2. Disagree
    3. Neither agree nor disagree
    4. Agree
    5. Strongly agree
14. I would not want to be friends with someone with Hepatitis B.
    1. Strongly disagree
    2. Disagree
    3. Neither agree nor disagree
    4. Agree
    5. Strongly agree
15. I would not employ someone with Hepatitis B to work for me.
    1. Strongly disagree
    2. Disagree
    3. Neither agree nor disagree
    4. Agree
    5. Strongly agree
16. I would feel uncomfortable having a conversation with someone who had Hepatitis B.
    1. Strongly disagree
    2. Disagree
    3. Neither agree nor disagree
    4. Agree
    5. Strongly agree
17. I would not kiss someone with Hepatitis B.
    1. Strongly disagree
    2. Disagree
    3. Neither agree nor disagree
    4. Agree
    5. Strongly agree
18. I would not date someone with Hepatitis B.
    1. Strongly disagree
    2. Disagree
    3. Neither agree nor disagree
    4. Agree
    5. Strongly agree
19. I would not marry someone with Hepatitis B.
    1. Strongly disagree
    2. Disagree
    3. Neither agree nor disagree
    4. Agree
    5. Strongly agree
20. I would avoid rooming with someone with Hepatitis B.
    1. Strongly disagree
    2. Disagree
    3. Neither agree nor disagree
    4. Agree
    5. Strongly agree

The following questions are about HIV and STI testing.

1. In the past 3-4 weeks, have you ever been tested for HIV (facility or self-testing)?
   1. Yes – In the past 3-4 weeks I’ve been tested and am HIV positive
   2. Yes – In the past 3-4 weeks I’ve been tested and am HIV negative
   3. Yes – In the past 3-4 weeks I’ve been tested and I never got my test results
   4. No – I have not tested for HIV in the past 3-4 weeks
2. Have you been tested for syphilis in the past 3-4 weeks?
   1. Yes
   2. No
3. What was the result of your syphilis test?
   1. Positive
   2. Negative
   3. Did not receive my result
4. Have you been tested for chlamydia in the past 3-4 weeks?
   1. Yes
   2. No
5. What was the result of your chlamydia test?
   1. Positive
   2. Negative
   3. Did not receive my result
6. Have you been tested for gonorrhea in the past 3-4 weeks?
   1. Yes
   2. No
7. What was the result of your gonorrhea test?
   1. Positive
   2. Negative
   3. Did not receive my result
8. Have you seen this image in the past 3-4 weeks? (Intervention one)
   1. Yes
   2. No
9. After viewing this image, did you like it, share it, or show it to friends or others?
   1. Yes
   2. No
10. Have you seen this image in the past 3-4 weeks? (Intervention three)
    1. Yes
    2. No
11. After viewing this image, did you like it, share it, or show it to friends or others?
    1. Yes
    2. No
12. Have you seen this video in the past 3-4 weeks? (Intervention two)
    1. Yes
    2. No
13. After viewing this video, did you like it, share it, or show it to friends or others?
    1. Yes
    2. No
14. Have you seen this video in the past 3-4 weeks? (Intervention four)
    1. Yes
    2. No
15. After viewing this video, did you like it, share it, or show it to friends or others?
    1. Yes
    2. No

**Follow-up Survey (Chinese)**

G1. 请问你的手机号码是多少？
（添加手机号码的目的仅为发放奖励与进行后续调查；整个过程中，我们都会严格保障参与者的隐私。）

a.（号码）

G2. 请问你的微信账号（非微信昵称）是多少？

（添加微信的目的仅为发放奖励与进行后续调查。我们的微信账号由一名主要研究者操作，其已接受严格的研究伦理培训；整个过程中，我们都会严格保障参与者的隐私。）

a.（账号）

下面的问题询问你有关乙肝检测的经历。

H1. 请问在过去3-4周内，你检测乙肝了吗? (即HBsAg检测)

1. 有
2. 没有

H2.你愿意提交乙肝检测结果的照片吗？（检测结果将被严格保密， 所有上传的检测结果将被保存在安全服务器中，且只有一位主要研究者可以查看。上传结果后，参与者将获得70元补贴。请确保年龄、性别、检测日期及检测结果信息清楚且正确。为了保护个人隐私，请使用遮盖或马赛克的方式，匿去检测者姓名。）

1. 愿意
2. 不愿意
3. 已经在微信提交

H3. 请问在过去3-4周内，你曾经跟医生讨论过关于乙肝检测的问题吗？

1. 有过
2. 没有

H4. 请问你是在哪里检测乙肝的？

1. 医院
2. 社区卫生服务中心／站
3. 私人诊所
4. 非政府组织
5. 疾病预防控制中心

H5. 请问你的乙肝检测结果是什么？

1. 目前感染乙肝（HBsAg+）
2. 以前感染过乙肝，但已治愈（抗-HBc+, 抗-HBs+, HBsAg-）
3. 以前打过疫苗（抗-HBs+, HBsAg-）
4. 没有打过疫苗，也没有被感染过（抗-HBs-, HBsAg）
5. 还没有拿到结果
6. 不清楚

H6. 请问，在拿到乙肝检测结果后， 你去看过肝炎专科医生吗？

1. 看过
2. 没有

H7. 请问你做过确认乙肝感染的检测吗？（即乙肝DNA检测）

1. 有
2. 没有

H8. 请问在过去3-4周内，你至少接种过一次乙肝疫苗吗？

1. 有过
2. 没有

H9. 请问检测后你没有上传乙肝检测报告单的理由是？

1. 担心泄露隐私
2. 已经做过检测，但还未拿到报告单
3. 报销补贴太少
4. 其他

H10. 请问你没有做乙肝检测的理由是？

1. 不知道去哪做检测
2. 没时间检测
3. 去检测时所需交通不方便
4. 检测费用太高
5. 不认为自己有感染的风险
6. 担心被检测出来乙肝会被歧视
7. 认为乙肝无法治愈
8. 不觉得乙肝是个严重的问题
9. 医生不让我做乙肝检测

下面的问题询问你有关丙肝检测的经历。

I1. 请问在过去3-4周内，你检测丙肝了吗? (即HCV抗体IgG检测)

a. 有

b. 没有

I2. 请问你愿意把丙肝检测报告单拍照并上传给我们吗？    
（检测结果将被严格保密， 所有上传的检测结果将被保存在安全服务器中，且只有一位主要研究者可以查看。上传结果后，参与者将获得40元补贴。请确保年龄、性别、检测日期及检测结果信息清楚且正确。为了保护个人隐私，请使用遮盖或马赛克的方式，匿去检测者姓名。）

a. 愿意（提交照片的链接）

b. 不愿意

c. 已经在微信提交

I3. 请问在过去3-4周内，你曾经跟医生讨论过关于丙肝检测的问题吗？

1. 有过
2. 没有

I4. 请问你是在哪里检测丙肝的？

1. 医院
2. 社区卫生服务中心／站
3. 私人诊所
4. 非政府组织
5. 疾病预防控制中心

I5. 请问你的丙肝检测结果是什么?

a. 阳性（HCV IgG+）

b. 阴性（HCV IgG-）

c. 还没有拿到结果

d． 不确定

I6. 请问在拿到丙肝检测结果后，你去看过肝炎专科医生吗？

1. 有过
2. 没有

I7. 请问你做过确认丙肝感染的检测吗？（即丙肝病毒载量检测）

1. 有过
2. 没有

I8. 请问丙肝病毒载量确认检测结果是什么？

1. 阳性
2. 阴性
3. 不确定

I9. 请问你没有上传丙肝检测报告单的理由是？

1. 担心泄露隐私
2. 已经做过检测，但还未拿到报告单
3. 报销补贴太少
4. 其他

I10. 请问你没有做丙肝检测的理由是？

a. 不知道去哪做检测

b. 没时间检测

c. 去检测时所需交通不方便

d. 检查费太贵

e. 不认为自己有感染的风险

f. 担心被检测出来丙肝会被歧视

g. 认为丙肝无法治愈

h. 不觉得丙肝是个严重的问题

i. 医生不让我做丙肝检测

以下问题将询问你对于乙肝检测的态度和经历，请回答你是否同意以下观点

J1. 为了保护大众，乙肝感染者应被隔离。

1. 非常不同意
2. 不同意
3. 既不同意也不反对
4. 同意
5. 非常同意

J2. 如果乙肝感染者从事与儿童密切接触的工作，他们可能会给儿童带来感染风险。

1. 非常不同意
2. 不同意
3. 既不同意也不反对
4. 同意
5. 非常同意

J3. 乙肝感染者不应被允许在餐馆等场所工作。

1. 非常不同意
2. 不同意
3. 既不同意也不反对
4. 同意
5. 非常同意

J4. 我觉得乙肝感染者很可怜。

1. 非常不同意
2. 不同意
3. 既不同意也不反对
4. 同意
5. 非常同意

J5. 乙肝感染者一定做过不好的事情，被感染是活该的。

1. 非常不同意
2. 不同意
3. 既不同意也不反对
4. 同意
5. 非常同意

J6. 孩子感染乙肝是因为父母的行为失当。

1. 非常不同意
2. 不同意
3. 既不同意也不反对
4. 同意
5. 非常同意

J7. 乙肝感染者应为他们的病痛感到羞耻。

1. 非常不同意
2. 不同意
3. 既不同意也不反对
4. 同意
5. 非常同意

J8. 乙肝感染者不干净。

1. 非常不同意
2. 不同意
3. 既不同意也不反对
4. 同意
5. 非常同意

J9. 如果我孩子的同学里有乙肝感染者，我不想再让孩子去那里上学。

1. 非常不同意
2. 不同意
3. 既不同意也不反对
4. 同意
5. 非常同意

J10. 我不想和乙肝感染者在同一间办公室工作。

1. 非常不同意
2. 不同意
3. 既不同意也不反对
4. 同意
5. 非常同意

J11. 如果隔壁小卖部的老板是乙肝感染者，我不想再去光顾了。

1. 非常不同意
2. 不同意
3. 既不同意也不反对
4. 同意
5. 非常同意

J12. 我穿乙肝感染者穿过的毛衣会觉得不舒服。

1. 非常不同意
2. 不同意
3. 既不同意也不反对
4. 同意
5. 非常同意

J13. 我和乙肝感染者一起吃饭会觉得不舒服。

1. 非常不同意
2. 不同意
3. 既不同意也不反对
4. 同意
5. 非常同意

J14. 我不想和乙肝感染者成为朋友。

1. 非常不同意
2. 不同意
3. 既不同意也不反对
4. 同意
5. 非常同意

J15. 我不想雇佣乙肝感染者。

1. 非常不同意
2. 不同意
3. 既不同意也不反对
4. 同意
5. 非常同意

J16. 我和乙肝感染者谈话会觉得不舒服。

1. 非常不同意
2. 不同意
3. 既不同意也不反对
4. 同意
5. 非常同意

J17. 我不会亲吻乙肝感染者。

1. 非常不同意
2. 不同意
3. 既不同意也不反对
4. 同意
5. 非常同意

J18. 我不会和乙肝感染者谈恋爱。

1. 非常不同意
2. 不同意
3. 既不同意也不反对
4. 同意
5. 非常同意

J19. 我不会和乙肝感染者结婚。

1. 非常不同意
2. 不同意
3. 既不同意也不反对
4. 同意
5. 非常同意

J20. 我会避免和乙肝感染者住在一起。

1. 非常不同意
2. 不同意
3. 既不同意也不反对
4. 同意
5. 非常同意

以下问题主要关于HIV和性传播疾病的检测

K1. 请问在过去3-4周内，你检测过HIV吗?

1. 有过 – 在过去3-4周内，我检测过HIV且感染了
2. 有过 – 在过去3-4周内，我检测过HIV且没有感染
3. 有过 – 在过去3-4周内，我不知道我的检测结果
4. 没有 – 在过去3-4周内，我没有检测过HIV

K2. 请问在过去3-4周内，你检测过梅毒吗？

1. 有过 - 在过去3-4周内， 我检测过梅毒
2. 没有 - 在过去3-4周内，我没有检测过梅毒

K3. 请问你的梅毒检测结果是什么？

1. 阳性
2. 阴性
3. 未收到检测结果

K4. 请问在过去3-4周内，你检测过衣原体吗？

1. 有过 - 在过去3-4周内， 我检测过衣原体
2. 没有 - 在过去3-4周内，我没有检测过衣原体

K5. 请问你的衣原体检测结果是什么？

1. 阳性
2. 阴性
3. 未收到检测结果

K6. 请问在过去3-4周内，你检测过淋病吗？

1. 有过 - 在过去3-4周内， 我检测过淋病
2. 没有 - 在过去3-4周内，我没有检测过淋病

K7. 请问你的淋病检测结果是什么？

1. 阳性
2. 阴性
3. 未收到检测结果

L1. 请问在过去3-4周内，你看过这幅漫画吗？（干预1）

1. 看过
2. 没看过

L2. 请问看过该漫画后，你是否给他点赞过，或是分享给自己的朋友等？

1. 是
2. 否

L3. 请问在过去3-4周内，你看过这幅漫画吗？（干预3）

1. 看过
2. 没看过

L4. 请问看过该漫画后，你是否给他点赞过，或是分享给自己的朋友等？

1. 是
2. 否

L5. 请问在过去3-4周内，你看过这个视频吗？（干预2）

1. 看过
2. 没看过

L6. 请问看过该视频后，你是否给他点赞过，或是分享给自己的朋友等？

1. 是
2. 否

L7. 请问在过去3-4周内，你看过这个视频吗？（干预4）

1. 看过
2. 没看过

L8. 请问看过该视频后，你是否给他点赞过，或是分享给自己的朋友等？

1. 是
2. 否
